# 863. ELISpot-BK virus assay for the assesment of BK virus cell-mediated immunity in kidney recipients

**DOI:** 10.1093/ofid/ofad500.908

**Published:** 2023-11-27

**Authors:** Sabina Herrera, Abiu Sempere, Natalia Egri, Javier Bernal Maurandi, Frederic Cofan, María Angeles Marcos, Mariona Pascal, Marta Bodro

**Affiliations:** Hospital Clinic, Barcelone, Catalonia, Spain; Hospital Clinic, Barcelone, Catalonia, Spain; Hospital Clinic, Barcelone, Catalonia, Spain; Hospital Clinic, Barcelone, Catalonia, Spain; Hospital Clinic, Barcelone, Catalonia, Spain; Hospital Clinic, Barcelone, Catalonia, Spain; Hospital Clinic, Barcelone, Catalonia, Spain; Hospital Clinic, Barcelone, Catalonia, Spain

## Abstract

**Background:**

The pre and post-transplant monitoring of BKV-specific T cells could be a useful marker to identify renal receptors at risk of BKV reactivation and consequently develop BKV nephropathy.

**Methods:**

We performed a prospective multicenter study between October 2020 and December 2021. BKV-ELISPOT was performed before and two months after kidney transplantation in included patients. Other variables such as demographic, immunosuppressive therapy, presence of biopsy-proven rejection, lymphopenia, CD4 count levels and development of BK infection and nephropathy were prospectively recorded.

**Results:**

We included 66 patients during the study period of whom median age was 56 (SD 16) and 60% were men. Twenty-nine percent of them received a prior transplantation, 17% was a living donor and 74% needed haemodialysis prior transplantation. Ninety-two percent received induction therapy and 86% maintenance therapy was with 3 drugs. Fifty-one percent of patients presented lymphopenia at 2 months post transplantation with a median CD4 count levels of 298 (IQR 176-899). Prior transplantation 30% of patients presented a reactive ELISPOT-BKV test, whereas at 2 months post transplantation only 21% of them tested positive. Five patients presented a BK infection and 2 of them a BK nephropathy, all of them presenting with impaired graft function at one year post transplantation.Twenty-eight percent of them presented a biopsy-proven acute rejection. All patients that presented a BKV infection presented a non-reactive ELISPOT-BKV test prior transplantation and at 2 months post transplantation. After performing multivariate analysis, neither lymphopenia, acute allograft rejection nor ELISPOT-BKV test were related to BKV infection, probably due to low incidence of BKV infection in this subgroup of kidney recipients.

**Conclusion:**

BKV-ELISPOT test could be a valuable tool to predict BKV infection or nephropathy and could be useful to implement preventive measures such pre-emptive reduction of immunosuppressive therapy in kidney recipients.

**Disclosures:**

**All Authors**: No reported disclosures

